# Real-World Data with CDK4/6 Inhibitors—A Single Center Experience from Croatia

**DOI:** 10.3390/jpm14090895

**Published:** 2024-08-23

**Authors:** Iva Skocilic, Marin Golcic, Anamarija Bukovica Petrc, Maja Kolak, Doris Kolovrat, Sanja Ropac, Jasna Marusic, Renata Dobrila-Dintinjana, Ivona Badovinac, Ani Mihaljevic Ferari, Ivana Mikolasevic

**Affiliations:** 1 Tumor Clinic, Clinical Hospital Center Rijeka, 51000 Rijeka, Croatia; iskocilic@gmail.com (I.S.); marin.golcic@gmail.com (M.G.); anamarija.bukovica@yahoo.com (A.B.P.); kolak.maja@gmail.com (M.K.); doris.kolovrat5@gmail.com (D.K.); sanjap0907@gmail.com (S.R.); renatadobrila@windowslive.com (R.D.-D.); ivona.badovinac@gmail.com (I.B.); amferari15@gmail.com (A.M.F.); 2 School of Medicine Rijeka, 51000 Rijeka, Croatia; 3 Department of Gastroenterology, Clinical Hospital Center Rijeka, 51000 Rijeka, Croatia

**Keywords:** cyclin-dependent kinase 4/6 inhibitors, progression-free survival, overall survival, adverse events

## Abstract

Background: There are limited real-world data (RWD) regarding the use of cyclin-dependent kinase (CDK) 4/6 inhibitors in western Balkan. The aim of our study was thus to analyze factors influencing progression-free survival (PFS) and overall survival (OS), along with the differences in adverse effects of CDK 4/6 therapy in a tertiary healthcare center in Croatia. Methods: We evaluated medical and demographic data for 163 consecutive patients with metastatic breast cancer treated with CDK4/6 inhibitors for at least one month, from October 2018, after the drug became available in Croatia. Eligible patients in our study were those patients who were treated with palbociclib, ribociclib, or abemaciclib. Results: The median PFS of CDK4/6 inhibitors treatment was 2.2 years (95% CI 1.8–3.3), with the longest ongoing treatment for 5.4 years. Treatment with CDK4/6 inhibitors in the first line was associated with a longer PFS compared to the second line or beyond (HR 0.50, 95% CI 0.3–0.9), and patients without liver metastasis exhibited longer survival compared to patients with liver metastasis (HR 0.46, 95% CI 0.2–0.8) (both *p* < 0.05). Regarding the choice of CDK4/6 inhibitors, ribociclib exhibited longer PFS compared to palbociclib (HR 0.49, 95% CI 0.29–0.82) (*p* = 0.0032), although the effect was not statistically significant when separating patients who were treated with CDK4/6 inhibitors in the first-line (HR 0.59, 95% CI 0.29–1.2), or second- or later-line therapy (0.49, 95% CI 0.15–1.55); the trend was present in both lines, however. The presence of liver metastasis (*p* = 0.04), initial luminal A grade (*p* = 0.039), and time to metastasis up to 5 years from the initial cancer (*p* = 0.002) were the only factors that remained statistically significant for PFS in multivariate analysis. Median OS since the diagnosis of metastatic disease was 4.5 years (95% CI 3.9–6.3), median OS since the start of CDK4/6 inhibitors treatment was 3.7 years (95% CI 3.4–4.4), while median OS from initial cancer diagnosis was 15.8 years (95% CI 13.8–18.3). There was no difference in OS based on the choice of CDK4/6 inhibitor (*p* = 0.44) or the adjuvant hormonal therapy (*p* = 0.12), although a nonsignificant trend for better OS with ribociclib was present for both regardless of whether it was in first- or second/later-line therapies (*p* > 0.05). In a multivariate analysis, only the presence of liver metastasis (*p* = 0.0003) and time to metastasis under 5 years from primary breast cancer (*p* = 0.03) were associated with a worse OS. Conclusions: Our study provides the RWD with the use of CDK4/6 inhibitors in the treatment of metastatic HR+/HER2− breast cancer. To our best knowledge, there are limited RWD regarding CDK 4/6 inhibitors use in western Balkan; thus, our study provides valuable data from everyday clinical practice for this region of Europe, bridging the gap between randomized clinical trials and clinical reality in western Balkan.

## 1. Introduction

Breast cancer is the most common cancer worldwide, accounting for 12.5% of all cancers and over 30% of all cancers in women [1]. More than 80% of invasive breast cancer cases are diagnosed in women over the age of 50, and 91% of deaths occur in this age group. Half of breast cancer deaths occur in women aged 70 or older [2]. Hormone receptor-positive (HR+), human epidermal growth factor receptor 2 (HER2)-negative cancer accounts for approximately 70% of all breast cancer cases, making it the most common subtype. The time to develop metastasis in luminal HER2-negative breast cancer can be variable, but these patients generally experience a longer disease-free interval compared to HER2-positive and triple-negative breast cancer patients. The most common sites of metastasis for luminal HER2-negative breast cancer are the bones, followed by the lungs, liver, and brain. Although it is considered the most favorable subtype in localized disease, metastatic HR+ tumors had a poor prognosis until the introduction of cyclin-dependent kinase (CDK) 4/6 inhibitors [3,4].

Endocrine therapy (ET) is the mainstay for the HR+ luminal subtype of breast cancer treatment, but its efficacy is limited by drug resistance, which is almost inevitable in advanced breast cancer patients. One of the basic biological features of malignant tumors is the uncontrolled proliferation and malignant transformation of tumor cells caused by a disruption of cell cycle regulation. Cyclin-dependent kinase 4/6 inhibitors restore the cell cycle by selectively inhibiting CDK 4 and 6 and blocking cell proliferation in a variety of tumor cells, including those of breast cancer [5].

The use of CDK4/6 inhibitors in combination with aromatase inhibitors or fulvestrant has fundamentally changed the treatment of ET resistance in hormone receptor-positive (HR+)/HER2-negative (HER2−) breast cancer [6,7,8]. Three CDK4/6 inhibitors are currently approved: palbociclib, ribociclib, and abemaciclib for first- and second-line treatment of HR+/HER2-metastatic breast cancer. The first approved CDK 4/6 inhibitor was palbociclib in 2015 following publication in the PALOMA-1/TRIO-18 study [9]. After that, the PALOMA-2 trial evaluated the efficacy of palbociclib in combination with letrozole versus letrozole alone in postmenopausal women with ER+/HER2− advanced breast cancer. The results showed a significant improvement in PFS for the palbociclib group (24.8 months) compared to the letrozole group (14.5 months) [10]. Additionally, the PALOMA-3 clinical trial showed similar results for the combination of ribociclib and fulvestrant [11]. MONALEESA-2, a phase III trial, assessed ribociclib in combination with letrozole versus letrozole alone in postmenopausal women with HR+/HER2− advanced breast cancer. The trial demonstrated a median PFS of 25.3 months for the ribociclib group compared to 16.0 months for the placebo group [12]. The MONALEESA-3 [13] and MONALEESA-7 trials [14] further confirmed the efficacy of ribociclib in combination with fulvestrant and endocrine therapy in both premenopausal and postmenopausal women, showing significant improvements in PFS. In 2017, the MONARCH 2 trial reported data evaluating abemaciclib in combination with fulvestrant in HR+/HER2- breast cancer patients who had progressed on prior endocrine therapy. The median PFS was 16.4 months for the abemaciclib plus fulvestrant group versus 9.3 months for the placebo plus fulvestrant group [15]. Finally, in the MONARCH 3 trial, abemaciclib was combined with a nonsteroidal aromatase inhibitor in postmenopausal women with HR+/HER2− advanced breast cancer. The median PFS was 28.2 months for the abemaciclib group compared to 14.8 months for the placebo group [16]. Individual studies on CDK 4/6 inhibitors show a similar effect on PFS, but different statistical significance for overall survival (OS) [16,17]. In general, CDK 4/6 inhibitors are well tolerated. Patients have a good quality of life, take peroral medication, and do not require frequent check-ups. The most common adverse events include anemia, neutropenia, fatigue, nausea, diarrhea, and QT prolongation [7,8]. Abemaciclib is structurally different from the other two CDK4/6 inhibitors and has greater selectivity for CDK4 compared to CDK6. CDK4 is particularly important for breast tumorigenesis, while CDK6 plays a crucial role in hematopoietic stem cell differentiation. Therefore, it shows a higher rate of diarrhea and fatigue, but a lower rate of hematologic adverse events, including neutropenia. In contrast to the current data for palbociclib, ribociclib has a higher incidence of QT interval prolongation [8]. Outside of the mentioned clinical studies, variable success of CDK 4/6 inhibitors therapy was reported in real-world data, which provides important insights regarding important clinical parameters such as PFS, OS, and adverse events in everyday clinical practice in patients with various demographic and clinical characteristics that usually are not adequately represented in randomized clinical studies [17,18,19].

CDK 4/6 inhibitors were approved by Croatia’s regulatory body in 2018 for the treatment of metastatic HR+ luminal breast cancer. The aim of our study was to analyze factors influencing PFS and OS, along with the differences in adverse effects, for CDK 4/6 inhibitor therapy in a tertiary healthcare center in Croatia since the introduction of CDK 4/6 inhibitors in Croatia in 2018. 

## 2. Methods

We conducted a retrospective observation study at the Clinical Hospital Center Rijeka, Croatia. We evaluated medical and demographic data for consecutive patients with metastatic breast cancer treated with CDK4/6 inhibitors for at least one month, from October 2018, after the drug became available in Croatia, until February 2024, in order to include at least one radiological scan. Eligible patients in our study were those patients who were treated with palbociclib, ribociclib, or abemaciclib. Patients with incomplete medical data or those with unknown clinical outcomes were excluded from this analysis.

The choice of CDK4/6 inhibitor and parallel hormonal therapy was based on the independent physician’s choice, although not all medications became available immediately. CDK4/6 inhibitors were approved for each patient in 12-week cycles, after which a regulatory body decided whether to allow further treatment based on clinical evaluation, radiological scan, and laboratory analyses. 

The study’s main goal was to evaluate factors influencing PFS (defined as the time from the start of CDK4/6 inhibitors to clinical or radiologic progression) and OS (defined as the time from the diagnosis of metastatic disease to death or loss of contact). Further analysis included adverse effects, graded according to Common Terminology Criteria for Adverse Events (CTCAE) version 4.0. In our study, adverse events were identified through medical history of each patient. 

Descriptive statistics were used to describe the data, the Chi-squared test was used to test the distributions, and the Mann–Whitney U-test was used to compare the groups. Spearman rank correlation was used for correlation analysis. Survival analyses were conducted using the Kaplan–Meier test, while univariate and multivariate analyses were performed using the Cox proportional hazard model. Multivariate analyses took into consideration the values which were found to be statistically significant in univariate analyses. Values of *p* <0.05 were considered statistically significant. Statistical analyses were performed using MedCalc Statistical Software, version 19 (MedCalc Software bvba, Ostend, Belgium).

The study was performed according to the Declaration of Helsinki. As the patient data were classified and the study was noninterventional and retrospective, signed informed consent was not required. 

## 3. Results

We included 163 patients, all women (100%). The median age at the time of initial cancer diagnosis was 57.0 (95% CI 54.6–59.0), ranging from 32.3 to 82.4 years, with the earliest primary cancer diagnosed in 1990 (Table 1).

The exact number of patients (7.4% (N = 12)) developed contralateral breast cancer later or a same-side local relapse. A total of 5 patients developed both contralateral breast cancer and same-side relapse (3.1%).

Contralateral breast cancer developed after a median of 9.1 years (95% CI 6.5–14.2) after the primary breast cancer diagnosis, while same-side relapse occurred after a median of 11.5 years (95% CI 6.3–14.2) after the primary. Synchronous breast cancer was found in 2.5% (N = 4) of patients.

For patients with known data, chemotherapy for primary cancer was applied on a median for 18 weeks (95% CI 18–21, ranging from 6 to 24 weeks) (Table 1). A total of 35 (21%) of patients received neoadjuvant chemotherapy and 63 (39%) received adjuvant chemotherapy. A similar number received regiments with or without paclitaxel (Table 1). Although all patients were recommended hormonal therapy, most commonly AI (N = 57, 35%), at least 20% (N = 32) did not complete the therapy.

When analyzing the original breast cancer, the median value of estrogen receptor expression was 90% (95% CI 87.4–90.0, ranging from 8 to 100), progesterone receptor 37.5% (95% CI 22.0–55.0, ranging from 0 to 100), and Ki67 23.0% (95% CI 21.4–25.0, ranging from 1 to 85).

The median age at which the metastatic cancer was discovered was 64.7 years (95% CI 62.1–66.5, ranging 33.5–84.8). A total of 25% of patients were younger than 55.5 years, and 25% of patients were older than 71.5 years. At the time of metastatic cancer diagnosis, the majority of patients were classified as either Eastern Cooperative Oncology Group (ECOG) 0 (N = 57, 35.0%) or ECOG 1 (N = 101, 62.0%), with 5 patients described as either ECOG 2 or 3 (3.0%).

Patients developed metastatic disease after a median of 4.3 years (95% CI 2.6–6.1) after primary breast cancer, although the range was 0–33.2 years, with the 75th percentile during the first 10.4 years (95% CI 8.4–11.9). The majority of patients initially reported with an early breast cancer (67.5% (N = 110)) compared to 53 patients (32.5%) initially diagnosed with metastatic breast cancer. Similarly, the majority of patients were postmenopausal at diagnosis (N = 124, 76.1%). 

Metastatic disease was discovered primarily after elevation of tumor markers in 44 patients (26.9%), clinical examination in 54 patients (33.1%), or routine scan in 26 patients (15.9%), while no data were available for 39 patients.

The most common sites of metastasis were the bones (N = 106, 65.0%), followed by lungs (N = 58, 35.6%) and lymph nodes (N = 39, 23.9%) (Table 2). A total of 79 patients had a cytological or histopathological confirmation of the metastatic lesion. The metastatic lesions had a median expression of estrogen receptors of 90% (95% 90–94.7, range 5–100), progesterone receptors of 0.45% (95% CI 0–5, range 0–100), and a median value of Ki67 of 35% (95% CI 26–37, range 10–80).

When compared to the initial cancer, the metastasis of the majority of patients expressed a higher level of estrogen receptors (N = 33, 61.1%). However, progesterone levels were lower in 60% of the patients (N = 30), and Ki67 levels were higher in 58.9% (N = 34) compared to the initial sample.

There was no difference between the ER expression (*p* = 0.37) between the initial and metastatic cancer, compared to the PR expression, which was lower in metastatic lesions, and Ki67 expression, which was higher (both *p* < 0.001).

A total of 160 patients were included in the analysis since there were missing data for 1 patient, and 2 patients ceased CDK4/6 treatment due to adverse effects in the first 6 months but reported no progression afterward. The majority of patients are still undergoing treatment (N = 81, 50.6%) (Table 3).

The median PFS of CDK4/6 treatment was 2.2 years (95% CI 1.8–3.3), with the longest ongoing treatment for 5.4 years. Treatment with CDK4/6 in the first line was associated with a longer PFS compared to the second line or beyond (HR 0.50, 95% CI 0.3–0.9), and patients without liver metastasis exhibited longer survival compared to patients with liver metastasis (HR 0.46, 95% CI 0.2–0.8) (both *p* < 0.05) (Table 4).

Although PFS did not differ based on the discovery type of the metastatic disease, there was a trend in longer PFS when the disease was discovered in asymptomatic patients compared to the discovery after elevation of tumor biomarkers (HR 0.50, 95% CI 0.25–0.97, *p* = 0.07). While previous same-side relapse was not associated with worse PFS, previous contralateral breast cancer was associated with a worse PFS (HR 2.2, 95% CI 0.8–5.7, *p* = 0.02) (Table 4).

Although age did not seem to affect PFS, there was a trend that patients younger than the age of 50 exhibited worse PFS compared to older patients (HR 1.68, 95% CI 0.82–3.47, *p* = 0.08). Although the trend was not statistically significant, compared to the youngest, the oldest cohort of patients exhibited an HR 0.28 (95% CI 0.08–1.00, *p* = 0.19) for PFS.

The relationship of time from initial diagnosis to metastatic disease showed to be a complex one, as patients with either synchronous metastatic disease or metastatic disease occurring 5 to 10 years after the initial diagnosis exhibited longer PFS when compared to patients with metastatic disease arising during the 0–5 years from initial diagnosis (HR 2.7 (95% CI 1.4–5.3) or more than 10 years after, HR 3.2 (95% CI 1.6–6.5), *p* = 0.0006, respectively).

Luminal subtype of the initial cancer was associated with a PFS, with patients with initial luminal stage A exhibiting a longer PFS (HR 0.47, 95% CI 0.3–0.8, *p* = 0.03) compared to luminal B patients. Initial tumor grade did not affect PFS, although there was a trend toward more prolonged survival with grade 1 compared to grades 2 and 3 (HR 0.51 and HR 0.61, but both *p* > 0.05) (Table 4).

For patients with a biopsied metastatic lesion, we noted there was no change in PFS regardless of the Ki67 value, or the change from initial cancer, although patients with estrogen receptors <90% in metastatic lesion exhibited worse PFS (HR 1.9, 95% CI 1.0–3.7, *p* = 0.02). Similarly, having progesterone receptors in a metastatic lesion higher than 10% was associated with a better PFS both compared to patients with a zero value (HR 0.32, 95% CI 0.13–0.8), or up to 10% (HR 0.37, 95% CI 0.2–0.7), *p* = 0.02 (Table 5).

Regarding the choice of CDK4/6, ribociclib exhibited longer PFS compared to palbociclib (HR 0.49, 95% CI 0.29–0.82) (*p* = 0.0032), although the effect was not statistically significant when separating patients who were treated with CDK4/6 inhibitors in the first-line (HR 0.59, 95% CI 0.29–1.2), or second or later-line therapy (0.49, 95% CI 0.15–1.55); the trend was present in both lines, however (Table 6).

The use of AI compared to SERD was associated with a longer PFS (HR 0.59, 95% CI 0.38–0.94), which was due to the longer survival in the first-line setting (HR 0.47, 95% CI 0.25–0.85), although the opposite was true in second-line and beyond (HR 2.99, 95% CI 0.95–9.3) (all *p* < 0.05).

A further multivariate analysis was undertaken, and only three factors remained statistically significant. The presence of liver metastasis (*p* = 0.04), initial luminal A grade (*p* = 0.039), and time to metastasis up to 5 years from the initial cancer (*p* = 0.002) were the only factors that remain statistically significant for PFS.

### 3.1. Overall Survival

Complete data for OS were reported for 162 patients. Median OS since the diagnosis of metastatic disease was 4.5 years (95% CI 3.9–6.3), median OS since the start of CDK4/6 treatment was 3.7 years (95% CI 3.4–4.4), while median OS from initial cancer diagnosis was 15.8 years (95% CI 13.8–18.3).

**Figure 1:**
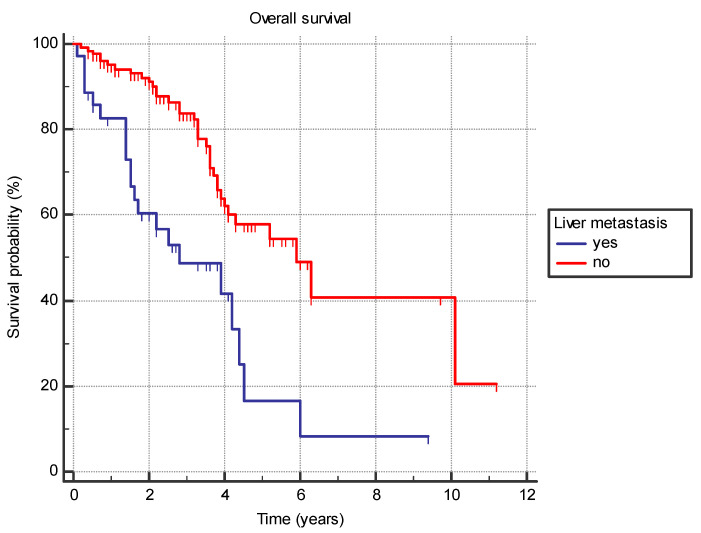
Overall survival in years based on the presence of the liver metastasis.

**Figure 2:**
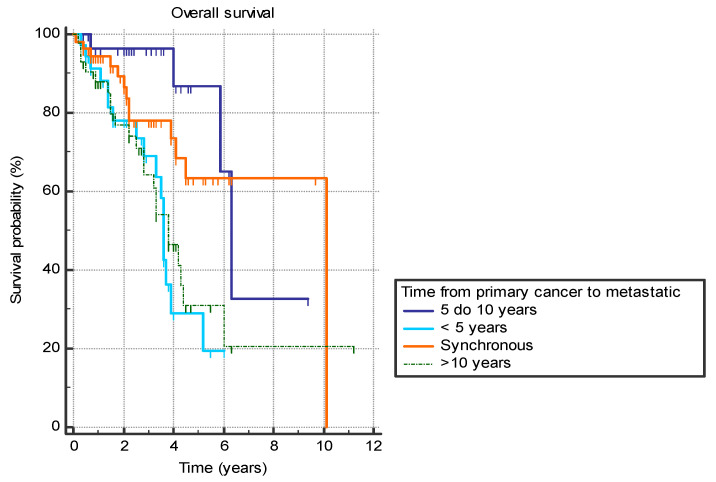
Overall survival in years based on the time between the primary breast cancer diagnosis and the diagnosis of the metastatic disease.

Several analyzed factors were associated with a difference in OS (Table 7). The presence of liver metastasis (HR 2.9 (95% CI 1.4–5.7), *p* = 0.0001) (Figure 1) or previously diagnosed contralateral breast cancer (HR 2.4 (95% CI 0.7–8.2), *p* = 0.03) was associated with worse OS. Furthermore, the time from the primary cancer to the appearance of metastatic disease also had a significant effect with bimodal distribution. Synchronous metastatic cancer or metastatic cancer diagnosed from 5 to 10 years after initial cancer both exhibited better OS compared to patients with metastatic diagnosis less than 5 years from primary (HR 0.39 (95% CI 0.2–0.9) and HR 0.19 (95% CI 0.1–0.4), respectively) or more than 10 years (HR 0.46 (95% CI 0.2–0.9) and HR 0.22 (95% CI 0.1–0.5), respectively) (*p* = 0.001) (Figure 2).

Evaluation of the metastatic site’s estrogen, progesterone, and Ki67 receptors also holds prognostic information for OS (Table 8). The majority of patients had Ki67 less than 30% (43%) (with the majority of patients having higher values compared to initial cancer (53%)), estrogen receptors below 90% (65%), and progesterone receptors 0 (49%). 

While Ki67 expression was not significant for PFS, having a Ki67 higher than 50% in a biopsied metastatic lesion was associated with a significantly shorter OS compared to patients with a Ki67 of 30–50% (HR 2.2 (95% CI 0.5–9.7) and less than 30% (HR 4.9 (95% CI 1.2–20.5)) (*p* = 0.004). Metastatic lesion Ki67 had a significant negative correlation with OS (r = −0.30, *p* = 0.02). 

While there was no difference for the OS when evaluating progesterone receptors as a whole group (*p* = 0.09), there was a difference inside the group. Expression of progesterone receptors of more than 10 was associated with a significantly longer OS compared to patients with a progesterone value of zero (HR 0.41, 95% CI 0.18–0.95, *p* = 0.049), while a nonsignificant trend was observed compared to patients with a receptor expression of 0–10.

There was no difference in OS based on the choice of CDK4/6 inhibitor (*p* = 0.44) or the adjuvant hormonal therapy (*p* = 0.12), although a nonsignificant trend for better OS with ribociclib was present in both regardless of whether it was in first- and second/later-line therapies (*p* > 0.05) (Table 9).

When evaluating factors associated with a poor prognosis for OS, we noticed that treatment with abemaciclib was associated with a longer OS compared to the other two agents (*p* = 0.03) for patients with liver metastasis. There was no difference in OS for other evaluated factors, although a nonsignificant trend was observed toward worse survival for the palbociclib arm in patients with metastatic cancer occurring more than 10 years after primary breast cancer (*p* = 0.08) (Table 10).

When evaluating the factors shown to affect OS in univariate analysis in a multivariate analysis, only the presence of liver metastasis (*p*= 0.0003) and time to metastasis under 5 years from primary breast cancer (*p* = 0.03) were associated with a worse OS.

### 3.2. Adverse Events

Most patients continued without dose reductions or discontinuations (91.5%, N = 130) at both 1- and 3-month periods, while 85.4% remained on the same dose and schedule at 6 months (N = 105). A dose reduction or change in CDK4/6 did not result in a shorter PFS (*p* = 0.24).

Details on adverse effects at three time points are given in Table 11, with leukopenia and neutropenia being the most common adverse effects. All recorded adverse events were most commonly Grade 1 or Grade 2. 

The adverse events were partially based on the choice of CDK4/6. Leukopenia and neutropenia were less common in abemaciclib than the other two agents (*p* < 0.05). On the other hand, diarrhea was typical for the abemaciclib group (*p* < 0.05), while nephrotoxicity was similar in abemaciclib and ribociclib but less common in palbociclib (*p* = 0.02) (Table 12). Line of treatment seemed to be important for the occurrence of adverse events as patients treated in first line with CDK4/6 had less leukopenia (*p* = 0.03), but not any of the other adverse events (all *p* > 0.05) compared to patients treated in second line or beyond.

The presence of adverse effects did not influence survival, either PFS or OS, although there was a trend in thrombocytopaenia at 1 month of treatment associated with a worse OS (*p* = 0.0502) (Table 13).

Rarer adverse effects include nausea and vomiting (5 patients at 1 month, 4 patients at 3 months, and 1 patient at 6 months), and pneumonitis (2 patients at 1 month, no patients at 3 months, and 1 patient at 6 months).

## 4. Treatment After CDK4/6

Out of the initial 160 patients with documented survival data, 81 patients are still undergoing CDK4/6 therapy (50.6%). Of 79 patients who progressed on CDK4/6 inhibitors, 63.3% (N = 50) were documented to have received a subsequent line of therapy, with 48 patients with reported survival data. The rest, 29 patients without a documented further line of treatment in our institution, exhibited a median OS of 0.1 years (95% CI 0.08–0.3) from the end of CDK4/6 therapy to death or loss of contact, with 2 patients with survival longer than 1 year (Table 14).

Patients who were treated with targeted treatment (everolimus or alpelisib) exhibited a longer PFS compared to patients treated with hormonal therapy (HR 0.33 (95% CI 0.15–0.74) or chemotherapy (HR 0.45 (95% CI 0.44–0.19–1.00)) (*p* = 0.012). There was a trend toward a longer PFS (HR 0.57, 95% CI 0.15–2.1) and OS (HR 0.68 (95% CI 0.13–3.41) favoring alpelisib; however, both values did not reach statistical significance (*p* = 0.41 and *p* = 0.65, respectively) (Table 15).

However, it was noted that patients who were treated with targeted treatment or chemotherapy in the later lines previously achieved a significantly longer PFS on CDK4/6 compared to patients later treated only with hormonal therapy (*p* = 0.005), suggesting that patients receiving hormonal therapy after CDK4/6 inhibitors had a worse response to previous treatment and could have been in a worse overall condition.

Patients who started further treatment following CDK4/6 inhibitors exhibited an OS of 1.4 years (95% CI 1.1–1.9) from the start of the next-line treatment to death or loss of contact. There was no difference in OS between the treatment choices, with a nonsignificant trend in more prolonged survival for targeted treatment (*p* = 0.62).

A total of 27 patients also started a second-line treatment following CDK4/6 progression, most commonly chemotherapy (59.2%, N = 16); 26 patients reported survival data. The median PFS on second-line treatment after CDK4/6 was 0.3 years (95% CI 0.2–0.5). There was no difference in survival based on the choice of agents.

The median OS for the patients after starting the second-line treatment following CDK4/6 progression was 0.8 years (95% CI 0.5–1.2), with no difference based on the choice of treatment (*p* = 0.61).

## 5. Discussion

There are limited real-world data regarding CDK 4/6 inhibitors use in western Balkan. To the best of our knowledge, previous real-world data studies included mostly palbociclib, and thus far, we have found limited real-world data, especially in this European region that compare the three CDK 4/6 inhibitors to each other. Our study showed that CDK4/6 inhibitors are effective and safe for patients with HR+/HER2− a/mBC, which is consistent with results seen in clinical trials.

Regarding the menopausal status, our group of patients has a high postmenopausal status of 79.4%, which is similar to what was reported in other real-world data studies [17,18,19,20]. In the MONALEESA and in the MONARCH study, the ECOG status was ≤1 while in PALOMA it was ≤2 [12,13,14,15,16]. Most of our patients had ECOG status between 0 and 1, while only four patients had ECOG status 2–3. In our study, 32.5% of patients were diagnosed with initially de novo metastatic disease, and others were previously treated as early breast cancer. The total percentage of patients (including adjuvant and neoadjuvant regimens) that were treated with prior chemotherapy in our cohort was 58.3%, while in MONARCH it was 39% and in PALOMA 48% [11,12,13,14,15,16]. Therefore, our patient population differs compared to registrational studies for CDK4/6 inhibitors [11,12,13,14,15,16], with patients at higher risk for disease progression. Twenty percent of patients developed metastatic disease during adjuvant hormonal treatment, and patients who developed metastatic disease developed it after a median of 4.3 years after primary breast cancer. Nearly half (48%) had pathological confirmation of metastatic disease with immunohistochemistry classification of tumor subtype. For patients with a biopsied metastatic lesion, we noted there was no change in PFS regardless of the Ki67 value, or the change from initial cancer. However, patients with estrogen receptors <90% in metastatic lesions exhibited worse PFS (HR 1.9, 95% CI 1.0–3.7, *p* = 0.02). Similarly, having progesterone receptors in a metastatic lesion higher than 10% was associated with a better PFS both compared to patients with a zero value (HR 0.32, 95% CI 0.13–0.8) or up to 10% (HR 0.37, 95% CI 0.2–0.7), *p* = 0.02. Considering the biological changes in breast cancer, and having a smaller number of patients who had metastatic lesions biopsied, we questioned whether the given treatment was the best option for those who were not biopsied.

Nevertheless, the PFS in our study was similar to RCT results, namely, the median PFS in our analysis for first-line CDK 4/6 inhibitors therapy was 26 months, which was similar to the published RCTs [9,10,11,12,13,14,15,16]. In our study, treatment with CDK4/6 inhibitors in the first line was associated with a longer PFS compared to the second line or beyond (HR 0.50, 95% CI 0.3–0.9), which is in alignment with data from the PRAEGNANT study, where it was shown that median PFS is significantly lower for those patients who received CDK 4/6 inhibitors in the second (8.7 months) or third line (4.7 months) in comparison to the first-line treatment (24.7 months) [21].

We wanted to determine which one of the CDK4/6 inhibitors is predominantly used in our region, considering that three of them have Croatia Health Insurance Fund (CHIF) approval. Although palbociclib was the first CDK 4/6 inhibitor that was introduced, in our study ribociclib was the most prescribed CDK 4/6 inhibitor. In other real-world data studies, palbociclib was the most prescribed medication [17,18,19,20]. In Croatia, palbociclib was introduced in 08/2018, ribociclib in 08/2018, and abemaciclib in 11/2019. In our study, ribociclib was the most prescribed CDK 4/6 inhibitor, and abemaciclib was the least prescribed because it was the last approved by the CHIF. Regarding the choice of CDK4/6, ribociclib exhibited longer PFS compared to palbociclib (HR 0.49, 95% CI 0.29–0.82) (*p* = 0.0032), although the effect was not statistically significant when separating patients who were treated with CDK4/6 inhibitors in the first line; the trend was present in both lines, however. 

In our CDK 4/6 inhibitor-treated patients, most were given letrozole (36.2%), followed by exemestane (12.3%), and anastrozole (8.6%) as complementary hormonal drugs in the first line of treatment. In the second line of treatment, Fulvestrant was prescribed to 41.7% of patients. The use of AI compared to SERD was associated with a longer PFS (HR 0.59, 95% CI 0.38–0.94), which was due to the longer survival in the first-line setting (HR 0.47, 95% CI 0.25–0.85), although the opposite was true in second line and beyond (HR 2.99, 95% CI 0.95–9.3) (all *p* < 0.05). 

Lastly, regarding the metastatic spread, in our study, there were a high proportion of patients with bone-only disease in 32.5% of cases, while in RTCs this was presented in 21–23% of patients [9,10,11,12,13,14,15,16]. Visceral metastasis was presented in 55.2% of our patients, which is similar to those data in the MONARCH-2 (53%) and PALOMA-2 (48%) studies [9,15]. However, in our study, only three factors had a statistically significant impact on PFS in multivariate analysis. The presence of liver metastasis (*p* = 0.04), initial luminal A grade (*p* = 0.039), and time to metastasis up to 5 years from the initial cancer (*p* = 0.002) were the only factors that remain statistically significant for PFS.

According to the meta-analysis published by Piezzo M et al. [22], OS data were available for the MONALEESA-2, MONALEESA-3, MONALEESA-7, MONARCH-2, PALOMA-1, and PALOMA-3 trials [11,13,15,23,24,25,26]. In this analysis, 2030 patients were receiving CDK 4/6 inhibitors while 1391 were receiving only endocrine therapy. The pooled HR of 0.763 (95% CI 0.683; 0.852) showed a significant reduction in the risk of dying for those cases that were treated with the CDK4/6 inhibitor (*p*-value < 0.0001) [22]. When the authors analyzed each CDK4/6 inhibitor, they found that a statistically significant reduction in the HR of dying was presented only for abemaciclib and ribociclib. On the other hand, they did not find a statistically significant reduction in the HR of dying for palbociclib [22].

OS in our study since the diagnosis of metastatic disease was 4.5 years (95% CI 3.9–6.3), median OS since the start of CDK4/6 treatment was 3.7 years (95% CI 3.4–4.4), while median OS from initial cancer diagnosis was 15.8 years (95% CI 13.8–18.3). There was no difference in OS based on the choice of CDK4/6 inhibitor (*p* = 0.44) or the adjuvant hormonal therapy (*p* = 0.12), although a nonsignificant trend for better OS with ribociclib was present regardless of whether it was in the first- and second/later-line therapies (*p* > 0.05). According to the MONALEESA-7 study, the estimated OS at 42 months was 70.2% in the ribociclib group and 46.0% in the placebo group [24,25]. In our study, when evaluating factors associated with a poor prognosis for OS, we noticed that treatment with abemaciclib was associated with a longer OS compared to the other two agents (*p* = 0.03) for patients with liver metastasis. There was no difference in OS for other evaluated factors, although a nonsignificant trend was observed toward worse survival for the palbociclib arm in patients with metastatic cancer occurring more than 10 years after primary breast cancer (*p* = 0.08) When evaluating the factors shown to affect OS in univariate analysis in a multivariate analysis, only the presence of liver metastasis (*p*= 0.0003) and time to metastasis under 5 years from primary breast cancer (*p* = 0.03) were associated with a worse OS.

In our data, most patients continued without dose reductions or discontinuations (91.5%) at both 1- and 3-month periods, while 85.4% remained on the same dose and schedule at 6 months. Thus, in our study, many fewer patients needed dose reduction and our results are more similar to other real-world data such as those of Ge I, et al. [17,26,27], where 23.3% of cases needed dose reduction, than in RCTs such as PALOMA where dose reduction was presented in 36% of patients. Adverse events were partially based on the choice of CDK4/6. Leukopenia and neutropenia were less common in abemaciclib than the other two agents (*p* < 0.05). On the other hand, diarrhea was typical for the abemaciclib group (*p* < 0.05), while nephrotoxicity was similar in abemaciclib and ribociclib but less common in palbociclib (*p* = 0.02). Thus, palbociclib could be a reasonable treatment option for those patients with a risk of kidney disease. Line of treatment seemed to be important for the occurrence of adverse events as patients treated in first line with CDK4/6 had less leukopenia (*p* = 0.03), but not any of the other adverse events (all *p* > 0.05) compared to patients treated in second line or beyond. In our study, dose reduction or change in CDK4/6 did not result in a shorter PFS (*p* = 0.24).

In our study, of patients who experienced disease progression on CDK4/6 inhibitors, 63.3% received a subsequent line of therapy. Although statistical significance was not reached, a longer PFS was achieved in those treated with alpelisib or everolimus compared to those treated with hormonal therapy (HR 0.33 (95% CI 0.15–0.74)) or chemotherapy (HR 0.45 (95% CI 0.44–0.19–1.00)) (*p* = 0.012). There was no effect on OS depending on the choice of therapy. Also, we noticed the worst response to subsequent therapy with treatment by only hormonal therapy, especially in a group of patients who had the longest response to CDK4/6 inhibitors. 

Finally, patients included in our study might not be representative of the broader population. Patients are not randomized to treatment groups, so it is difficult to establish causality. There is also more heterogeneity in prior treatment, patient comorbidities, and treatment partners of CDK 4/6 inhibitors, which complicates the interpretation of the results. Follow-up is shorter than desired, which affects observation of long-term outcomes. However, our data show no major differences compared to RCTs except for shorter OS. This could be explained by having more patients receiving neoadjuvant and adjuvant chemotherapy for early-stage breast cancer, indicating initially more aggressive disease, having patients with poorer performance status, and shorter follow-up time. In our study, while multiple factors are associated with a difference in PFS and OS, liver metastasis and the time from initial cancer to metastatic disease under 5 years were shown to be the most consistent and negative prognostic factors. Our study provides the RWE data with the use of CDK4/6 inhibitors in the treatment of metastatic HR+/HER2− breast cancer. To our best knowledge, there are limited real-world data regarding CDK 4/6 inhibitors use in western Balkan, thus our study provides valuable data from everyday clinical practice for this region of Europe, bridging the gap between RCTs and clinical reality in western Balkan. In the future, it would be of great importance for collaboration among several oncology clinics from this region of Europe to collect multicenter data on a larger group of patients.

## Figures and Tables

**Table 1:** Data regarding the initial breast cancer diagnosis.

Parameter	Number of Patients	Percentage of Patients
Initial T stage		
1	38	23.3
2	50	30.7
3	29	17.8
4	34	20.9
Not reported	12	7.4
Initial N stage		
0	36	22.1
1	71	43.6
2	34	20.9
3	11	6.7
Not reported	11	6.7
Initial M stage		
No	110	67.5
Yes	53	32.5
Initial cancer grade		
1	14	8.6
2	120	73.6
3	14	8.6
Not reported	15	9.2
Luminal breast cancer		
A	30	18.4
B	102	62.6
Not reported	31	19.0
Contralateral breast cancer		
No	12	7.4
Yes	151	92.6
Same-side relapse		
No	12	7.4
Yes	151	92.6
Neoadjuvant chemotherapy		
No	97	59.5
Yes	35	21.5
Not reported	31	19.0
Adjuvant chemotherapy		
No	69	42.3
Yes	63	38.7
Not reported	31	19.0
Type of chemotherapy		
With paclitaxel	34	20.9
AC + weekly paclitaxel	30	18.4
Paclitaxel	3	1.8
FAC + paclitaxel	1	0.6
Without paclitaxel	45	27.6
AC	11	6.7
CMF	2	1.2
FAC	32	19.6
Not reported	5	3.1
Completed hormonal therapy for primary		
No	32	19.6
Yes	101	62.0
Neoadjuvant	1	0.6
Not reported	29	17.8
Type of hormonal therapy		
Not reported or did not use	64	39.3
SERM	27	16.6
SERM/AI	15	9.2
AI	57	35.0
Anastrozole	42	25.8
Letrozole	21	12.9
Exemestane	8	4.9
Unknown AI	1	0.6

AC = doxorubicin + cyclophosphamide, AI = aromatase inhibitor, CDK = cyclin-dependent kinase, CMF = cyclophosphamide + methotrexate + 5-fluorouracil, FAC = 5-fluorouracil + doxorubicin + cyclophosphamide, SERD = selective estrogen receptor degrader, SERM = selective estrogen receptor modulator. Bold values denote statistical significance.

**Table 2:** Data on histopathological evaluation of metastatic lesions.

Parameter	Number of Patients	Percentage
Successful cytological or histopathological confirmation of the disease		
No	84	51.5
Yes	79	48.5
Bone	16	9.8
Lymph node	11	6.7
Ascites/pleural fluid	4	2.5
Skin or subcutaneous lesion	14	8.6
Visceral organ	34	20.9
ER in metastatic lesion compared to initial cancer		
Higher	33	61.1
The same	9	16.7
Lower	12	22.2
PR in metastatic lesion compared to initial cancer		
Higher	7	14.0
The same	13	26.0
Lower	30	60.0
Ki67 in metastatic lesion compared to initial cancer		
Higher	31	53.4
The same	11	19.0
Lower	16	27.6
Distribution of the most common metastatic sites		
Bone	106	65.0
Lungs	58	35.6
Lymph nodes	39	23.9
Liver	35	21.5
Skin, subcutaneous tissue or muscle	14	8.6
Peritoneum	6	3.7
Pleura	6	3.7
Ovaries	6	3.7
Brain	4	2.5
Adrenal glands	3	1.8
Leptomeningeal	1	0.6
Not all data are available for each patient.		

Progression-free survival (PFS).

**Table 3:** Details regarding the use of CDK4/6 inhibitors in metastatic disease.

Parameter	Number of Patients	Percentage (%)
CDK4/6 type of medication		
Abemaciclib	36	22.1
Ribociclib	66	40.5
Palbociclib	57	35.0
Dual (switched)	3	1.8
Triple (switched)	1	0.6
CDK4/6 line of treatment		
First	130	79.8
Second	22	13.5
Third	9	5.5
Fourth	2	1.2
Complementary hormonal drug		
SERD (Fulvestrant)	68	41.7
SERD/AI switch	1	0.6
Not reported	1	0.6
AI	94	57.1
Anastrozole	14	8.6
Exemestan	20	12.3
Letrozole	59	36.2

AI = aromatase inhibitor, CDK = cyclin-dependent kinase, SERD = selective estrogen receptor degrader. Bold values denote statistical significance.

**Table 4:** Factors associated with PFS in metastatic disease.

Parameter	Number of Patients (%)	PFS, Years (95% CI)	*p*-Value
ECOG status			
0	57	2.5 (1.8–3.4)	
1	99	2.1 (1.6–3.5)	
2 or 3	4	3.0 (0.5–3.0)	0.68
Line of treatment			
First	127	3.0 (2.0–3.6)	
Second or later	33	1.5 (0.9–2.0)	***p* = 0.0037**
Metastatic site			
Bone metastasis	104	2.1 (1.6–3.3)	
No bone metastasis	56	2.3 (1.8–3.3)	*p* = 0.25
Lung metastasis	55	2.0 (1.7–2.9)	
No lung metastasis	105	2.5 (1.6–3.5)	*p* = 0.67
Liver metastasis	35	1.5 (0.6–2.1)	
No liver metastasis	125	3.0 (1.9–4.4)	***p* = 0.001**
Lymph nodes metastasis	39	3.0 (1.7–3.3)	
No lymph node metastasis	121	2.1 (1.7–3.3)	*p* = 0.44
Skin/subcutaneous metastasis	14	2.9 (0.9–2.9)	
No skin/subcutaneous metastasis	146	2.1 (1.8–3.3)	*p* = 0.90
Peritoneum metastasis	6	1.8 (0.8–1.8)	
No peritoneal metastasis	154	2.3 (1.8–3.3)	*p* = 0.84
Pleural metastasis	6	2.9 (2.9–2.9)	
No pleural metastasis	154	2.1 (1.7–3.3)	*p* = 0.12
Ovarian metastasis	6	Not reached	
No ovarian metastasis	154	2.2 (1.9–3.3)	0.25
Menopausal status			
Premenopausal	39	2.0 (1.2–3.0)	
Postmenopausal	121	2.3 (1.7–3.4)	0.17
Discovery of metastatic disease			
Elevation of tumor markers	43	1.8 (0.9–2.7)	
Asymptomatic scan or lab	26	n/r	
Clinical suspicion	53	1.8 (1.4–3.3)	0.16
Previous contralateral tumor			
Yes	12	1.3 (0.5–3.4)	
No	148	2.4 (1.8–3.3)	**0.02**
Previous same-side relapse			
Yes	12	1.75 (0.4–2.4)	
No	148	2.2 (1.8–3.3)	0.39
Synchronous primary			
Yes	4	1.6 (0.8–1.6)	
No	156	2.2 (1.8–3.3)	0.77
Initially metastatic disease			
No	108	2.0 (1.5–2.9)	
Yes	52	Not reached	**0.04**
Previous chemotherapy			
No	70	3.0 (1.8–3.3)	
Yes	90	1.9 (1.5–3.3)	0.11
Without paclitaxel	42	2.1 (1.5–3.6)	
With paclitaxel	34	1.7 (0.9–3.4)	0.39
Previous hormonal therapy			
AI	56	1.9 (1.45–3.4)	
SERM	27	2.4 (1.2–3.5)	
SERM/AI	15	1.3 (0.4–1.8)	0.24
Age of metastatic disease (median)			
Younger than 64.7 years	80	2.0 (1.75–2.9)	
Older than 64.7 years	80	3.3 (1.7–3.4)	0.24
Age of metastatic disease (category)			
50 or younger	22	1.2 (0.9–3.0)	
50–59 years	39	2.2 (1.8–3.5)	
60–69 years	51	3.3 (1.5–4.4)	
70–79 years	41	2.3 (1.6–3.3)	
80 or older	7	Not reached	0.35
Time from original cancer to metastatic cancer			
Synchronous	52	Not reached	
Less than 5 years	36	1.5 (0.6–2.1)	
5 to 10 years	31	4.4 (2.4–4.4)	
More than 10 years	40	1.8 (1.2–3.3)	**0.0006**
Initial T stage			
1	37	1.6 (0.8–3.3)	
2	50	2.3 (1.5–3.4)	
3	29	3.6 (2.0–3.5)	
4	34	2.5 (1.8–3.4)	0.11
Initial N stage			
0	35	3.3 (0.7–3.6)	
1	70	2.5 (1.6–3.4)	
2	34	2.0 (1.6–2.3)	
3	11	Not reached	0.59
Initial luminal differentiation			
A	30	Not reached	
B	70	2.0 (1.7–3.3)	**0.03**
Initial cancer grade			
1	14	Not reached	
2	117	2.2 (1.7–3.3)	
3	14	2.0 (1.5–2.0)	0.33

AI = aromatase inhibitor, CDK = cyclin-dependent kinase, PFS = progression-free survival, SERM = selective estrogen receptor modulators, SERD = selective estrogen receptor degrader. Bold values denote statistical significance.

**Table 5:** Difference in PFS depending on the expression of the receptors on the metastatic site biopsy.

Pathological Expression	Number of Patients (%)	OS, Years (95% CI)	*p*-Value
Ki67 levels			
Up to 30%	26	1.8 (0.9–3.6)	
30 to 50%	25	1.45 (1.3–3.3)	
50% or higher	11	0.9 (0.2–2.0)	0.73
Change in Ki67 from the initial cancer			
The same	10	3.3 (0.8–3.3)	
Negative change	16	1.75 (0.9–2.0)	
Rise up to 20%	17	3.6 (0.6–3.6)	
Rise over 20%	14	1.8 (0.9–2.3)	0.46
Estrogen receptors			
>90%	47	2.1 (1.45–3.6)	
< 90%	26	1.3 (0.6–2.0)	**0.019**
Progesterone receptors			
Zero	34	1.45 (0.9–2.3)	
Less than 10	12	1.2 (0.6–3.5)	
More than 10	22	Not reached	**0.02**

PFS = progression-free survival. Bold values denote statistical significance.

**Table 6:** Difference in PFS depending on the choice of CDK4/6 treatment and adjuvant hormonal therapy.

Medication	Number of Patients	PFS (95% CI), Years	*p*-Value
CDK4/6			
Abemaciclib	36	2.7 (1.6–3.6)	
Palbociclib	57	1.7 (1.2–2.0)	
Ribociclib	63	3.3 (2.0–4.4)	
Dual or triple-switch	4	Not reached	***p* = 0.0032**
Adjuvant hormonal therapy			
AI	91	3.3 (2.0–4.4)	
Fulvestrant	67	1.7 (1.3–2.4)	**0.019**

AI = aromatase inhibitor, CDK = cyclin-dependent kinase, PFS = progression-free survival. Bold values denote statistical significance.

**Table 7:** Factors associated with OS in metastatic disease.

Parameter	Number of Patients (%)	OS, Years (95% CI)	*p*-Value
ECOG status			
0	57	6.0 (4.3–10.1)	
1	101	3.9 (3.6–5.9)	
2 or 3	4	Not reached	0.19
Line of treatment			
First	129	Not reached	
Second or later	33	4.5 (4.1–10.1)	0.52
Metastatic site			
Bone metastasis	105	5.2 (4.0–10.1)	
No bone metastasis	56	4.4 (3.2–6.0)	0.52
Lung metastasis	57	6.0 (3.3–6.0)	
No lung metastasis	105	4.5 (3.9–10.1)	0.94
Liver metastasis	35	2.8 (1.6–4.4)	
No liver metastasis	127	5.9 (4.1–10.1)	**0.0001**
Lymph nodes metastasis	39	6.0 (3.3–6.0)	
No lymph node metastasis	123	4.4 (3.8–10.1)	0.64
Skin/subcutaneous metastasis	14	Not reached	
No skin/subcutaneous metastasis	148	4.5 (3.9–6.3)	0.88
Peritoneum metastasis	6	3.3 (3.2–3.3)	
No peritoneal metastasis	156	4.5 (4.0–6.3)	0.32
Pleural metastasis	6	Not reached	
No pleural metastasis	156	4.4 (3.9–6.3)	0.17
Ovarian metastasis	6	Not reached	
No ovarian metastasis	156	4.4 (3.9–6.3)	0.82
Menopausal status			
Premenopausal	39	4.1 (3.6–6.0)	
Postmenopausal	123	5.9 (3.9–10.1)	0.34
Discovery of metastatic disease			
Elevation of tumor markers	44	3.7 (3.3–4.2)	
Asymptomatic scan or lab	26	5.2 (4.3–5.2)	
Clinical suspicion	53	4.5 (3.8–6.3)	0.11
Previous contralateral tumor			
Yes	12	3.2 (2.2–3.8)	
No	150	5.2 (4.1–10.1)	**0.03**
Previous same-side relapse			
Yes	12	3.8 (1.7–6.3)	
No	150	5.2 (3.9–10.1)	0.18
Synchronous primary			
Yes	4	3.2 (n/r)	
No	158	4.5 (3.9–6.3)	0.85
Initially metastatic disease			
No	109	4.2 (3.6–5.9)	
Yes	53	10.1 (4.5–n/r)	0.09
Previous chemotherapy			
No	72	6.3 (3.8–10.1)	
Yes	90	4.3 (3.8–6.0)	0.43
Without paclitaxel	43	5.9 (3.9–6.0)	
With paclitaxel	34	3.8 (3.5–5.2)	0.34
Previous hormonal therapy			
AI	56	4.4 (3.3–6.3)	
SERM	27	3.7 (3.5–5.9)	
SERM/AI	15	3.3 (2.8–4.0)	0.36
Age of metastatic disease (median)			
Younger than 64.7 years	81	5.2 (3.9–6.3)	
Older than 64.7 years	81	4.5 (3.8–4.5)	0.70
Age of metastatic disease (category)			
50 or younger	22	4.1 (2.2–10.1)	
50–59 years	39	4.4 (3.7–6.3)	
60–69 years	52	6.0 (3.9–6.0)	
70–79 years	41	Not reached	
80 or older	8	Not reached	0.81
Time from original cancer to metastatic cancer			
Synchronous	53	10.1 (4.5–n/r)	
Less than 5 years	36	3.6 (3.3–5.2)	
5 to 10 years	31	6.3 (5.0–8.8)	
More than 10 years	41	3.8 (2.8–4.4)	**0.001**
Initial cancer T stage			
1	38	3.9 (2.8–6.0)	
2	50	59 (3.9–5.9)	
3	29	Not reached	
4	34	10.1 (5.2–n/r)	0.19
Initial cancer N stage			
0	36	3.9 (3.6–3.9)	
1	70	5.2 (3.9–10.1)	
2	34	4.2 (2.8–4.2)	
3	11	5.9 (n/r)	0.69
Initial luminal differentiation			
A	30	4.5 (3.6–4.5)	
B	102	4.4 (3.8–5.2)	0.57
Initial cancer grade			
1	14	Not reached	
2	119	4.5 (3.9–10.1)	
3	14	2.8 (2.2–2.8)	0.79

AI = aromatase inhibitor, CDK = cyclin-dependent kinase, OS = overall survival, SERM = selective estrogen receptors modulators, SERD = selective estrogen receptor degrader. Bold values denote statistical significance.

**Table 8:** Difference in OS depending on the expression of the receptors on the metastatic site biopsy.

Pathological Expression	Number of Patients (%)	OS, Years (95% CI)	*p*-Value
Ki67 levels			
Up to 30%	27 (43)	4.4 (3.9–6.0)	
30 to 50%	25 (40)	3.3 (1.7–6.3)	
50% or higher	11 (17)	1.4 (0.5–2.8)	**0.0043**
Change in Ki67 from the initial cancer			
The same	11 (19)	Not reached	
Negative change	16 (28)	3.9 (2.8–4.4)	
Rise up to 20%	17 (29)	Not reached	
Rise over 20%	14 (24)	2.8 (1.5–6.0)	0.51
Oestrogen receptors			
>90%	26 (35)	4.2 (2.8–6.3)	
Below 90%	48 (65)	3.8 (1.5–6.0)	0.35
Progesterone receptors			
Zero	34 (49)	3.9 (2.8–4.3)	
Less than 10	13 (19)	3.7 (2.5–4.4)	
More than 10	22 (32)	6.0 (4.0–6.0)	0.09

OS = overall survival. Bold values denote statistical significance.

**Table 9:** Difference in OS depending on the choice of CDK4/6 treatment and adjuvant hormonal therapy.

Medication	Number of Patients	OS (95% CI), Years	*p*-Value
CDK4/6			
Abemaciclib	36	3.9 (3.6–3.9)	
Palbociclib	57	4.3 (3.5–5.9)	
Ribociclib	65	Not reached	0.44
Adjuvant hormonal therapy			
AI	93	6.0 (4.0–6.0)	
Fulvestrant	67	4.2 (3.3–6.3)	0.12

AI = aromatase inhibitor, CDK = cyclin-dependent kinase, OS = overall survival. Bold values denote statistical significance.

**Table 10:** Difference in OS depending on the choice of CDK4/6 treatment in first-line therapy, based on factors associated with a worse OS.

	Abemaciclib	Palbociclib	Ribociclib	*p*
Liver metastasis	3.9 (2.8–n/r)	1.4 (0.5–1.7)	1.6 (0.7–1.6)	**0.03**
Previous contralateral cancer	n/r	3.8 (0.5-n/r)	2.2 (2.2–2.5)	0.41
Time to metastatic disease <5 years from primary	3.9 (3.6–3.9)	n/r	2.8 (1.1–3.7)	0.49
Time to metastatic disease >10 years from primary	n/r	1.7 (0.5–3.8)	3.8 (2.2–3.9)	0.08
Ki67 50% or higher	1.4 (0.7–n/r)	n/r	0.5 (0.3–2.8)	0.65
Progesterone <10%	3.9 (1.4–3.9)	1.5 (0.5–1.5)	3.7 (1.5–3.8)	0.73

Bold values denote statistical significance.

**Table 11:** Presence of adverse effects on CDK4/6 treatment at different time points after CDK4/6 initiation.

Presence of Adverse Effect	Time Point					
	1 Month		3 Months		6 Months	
	No. of Pts.	Perc. of Pts.	No. of Pts.	Perc. of Pts.	No. of Pts.	Perc. of Pts.
Leukopenia						
No	80	53%	83	60%	57	50%
Yes	70	47%	55	40%	56	50%
Grade 1	53	35%	40	29%	41	36%
Grade 2	14	9%	15	11%	15	13%
Grade 3	3	2%	0	0%	0	0%
Neutropenia						
No	56	37%	69	50%	45	40%
Yes	94	63%	69	50%	68	60%
Grade 1	38	25%	29	21%	24	21%
Grade 2	40	27%	29	21%	33	29%
Grade 3	15	10%	11	8%	11	10%
Thrombocytopenia						
No	140	93%	133	96%	105	93%
Yes	10	7%	5	4%	8	7%
Grade 1	7	5%	2	1%	6	5%
Grade 2	1	1%	3	2%	2	2%
Grade 3	2	1%	0	0%	0	0%
Anemia						
No	131	87%	113	82%	102	90%
Yes	19	13%	25	18%	11	10%
Grade 1	17	11%	21	15%	10	9%
Grade 2	2	1%	4	3%	0	0%
Grade 3	0	0%	0	0%	1	1%
Diarrhea						
No	135	90%	128	93%	105	93%
Yes	15	10%	10	7%	8	7%
Grade 1	9	6%	8	6%	4	4%
Grade 2	5	3%	2	1%	3	3%
Grade 3	1	1%	0	0%	1	1%
Nephrotoxicity						
No	139	93%	132	96%	107	95%
Yes	11	7%	6	4%	6	5%
Grade 1	6	4%	2	1%	5	4%
Grade 2	4	3%	2	1%	1	1%
Grade 3	1	1%	2	1%	0	0%
Hepatotoxicity						
No	142	95%	128	93%	105	93%
Yes	8	5%	10	7%	8	7%
Grade 1	4	3%	5	4%	7	6%
Grade 2	2	1%	2	1%	1	1%
Grade 3	2	1%	3	2%	0	0%

CDK = cyclin-dependent kinase.

**Table 12:** Relationship between the choice of CDK4/6 inhibitors and adverse effects at 1 month.

Patients	Patients on Abemaciclib	Patients on Palbociclib	Patients on Ribociclib	*p*-Value
Leukopenia				
Number of patients	9	32	29	
Percentage of patients	33.3	58.2	52.3	**0.** **0055**
Neutropenia				
Number of patients	14	39	40	
Percentage of patients	38.9	70.9	72.7	**0.002**
Thrombocytopenia				
Number of patients	1	3	6	
Percentage of patients	2.8	5.4	10.9	0.28
Anemia				
Number of patients	8	3	8	
Percentage of patients	22.2	5.4	14.5	0.06
Diarrhea				
Number of patients	12	1	0	
Percentage of patients	33.3	1.8	0	**<0.0001**
Nephrotoxicity				
Number of patients	5	0	6	
Percentage of patients	13.9	0	10.9	**0.02**
Hepatotoxicity				
Number of patients	4	2	2	
Percentage of patients	11.1	3.6	3.6	0.23

CDK = cyclin-dependent kinase. Bold values denote statistical significance.

**Table 13:** Difference in survival depending on adverse effects 1 month following CDK4/6 treatment.

Presence of Adverse Effect	PFS (95% CI), Years	*p*	OS (95% CI), Years	*p*
Leukopenia				
No	2.3 (1.8–3.4)		4.3 (3.6–6.3)	
Yes	1.8 (1.3–3.3)	0.32	5.9 (3.7–10.1)	0.43
Neutropenia				
No	2.4 (1.75–3.6)		4.4 (4.1–6.3)	
Yes	1.8 (1.4–3.0)	0.63	4.2 (3.7–10.1)	0.79
Thrombocytopenia				
No	2.1 (1.75–3.0)		4.5 (3.8–6.3)	
Yes	1.45 (0.9–1.8)	0.19	3.9 (1.5–4.2)	0.05
Anemia				
No	2.0 (1.6–3.3)		4.3 (3.8–6.0)	
Yes	2.2 (1.3–2.7)	0.82	10.1 (2.8–n/r)	0.92
Diarrhea				
No	2.0 (1.65–2.9)		4.3 (3.8–6.0)	
Yes	2.3 (1.6–3.6)	0.91	10.1 (3.9–n/r)	0.28
Nephrotoxicity				
No	2.0 (1.65–2-7)		4.3 (3.8–6.0)	
Yes	3.4 (0.9–3.4)	0.71	Not reached	0.86
Hepatotoxicity				
No	2.0 (1.7–2.9)		4.4 (3.8–6.0)	
Yes	2.7 (0.1–3.4)	0.29	3.6 (0.3–3.6)	0.39

OS = overall survival, PFS = progression-free survival. Bold values denote statistical significance.

**Table 14:** Type of treatment following CDK4/6 progression.

	First Line		Second Line	
Type of Medication	No.	%	No.	%
Targeted treatment	23	45%	2	7%
Alpelisib	11	22%	2	7%
Everolimus	12	24%	0	0%
Hormonal treatment	16	31%	9	33%
AI	7	14%	6	22%
SERD	5	10%	2	7%
SERM	4	8%	1	4%
Chemotherapy	12	24%	16	59%
CMF	2	4%	1	4%
Gemcitabine	1	2%	0	0%
Capecitabine	5	10%	7	26%
Paclitaxel	4	8%	6	22%
Vinorelbine	0	0%	1	4%
Carboplatin	0	0%	1	4%

AI = aromatase inhibitor, CDK = cyclin-dependent kinase, CMF = cyclophosphamide, methotrexate, 5-fluorouracil, SERM = selective estrogen receptors modulators, SERD = selective estrogen receptor degrader.

**Table 15:** Survival following progression on CDK4/6 inhibitors based on the type of medication used.

First Line After CDK4/6 Progression				
Type of Medication	PFS (95% CI), Years	*p*-Value	OS (95% CI), Years	*p*-Value
Targeted treatment	1.0 (0.5–1.3)		Not reached	
Hormonal treatment	0.4 (0.2–0.5)		1.4 (0.5–2.7)	
Chemotherapy	0.3 (0.2–0.7)	**0.012**	1.1 (0.9–1.9)	0.61
Second line after CDK4/6 progression				
Targeted treatment	0.6 (06–2.2)		0.85 (0.85–0.85)	
Hormonal treatment	0.2 (0.2–0.3)		0.6 (0.3–1.1)	
Chemotherapy	0.3 (0.3–0.5)	0.13	0.8 (0.5–1.2)	0.61

OS = overall survival, PFS = progression-free survival. Bold values denote statistical significance.

## Data Availability

The original contributions presented in the study are included in the article, further inquiries can be directed to the corresponding authors.

## References

[1] Wang X., Zhao S., Xin Q., Zhang Y., Wang K., Li M. (2024). Recent progress of CDK4/6 inhibitors’ current practice in breast cancer. Cancer Gene Ther..

[2] Giaquinto A.N., Sung H., Miller K.D., Kramer J.L., Newman L.A., Minihan A., Jemal A., Siegel R.L. (2022). Breast Cancer Statistics, 2022. CA Cancer J. Clin..

[3] Gehrchen M.L., Berg T., Garly R., Jensen M.B., Eßer-Naumann S., Rønlev J.D., Nielsen H.M., Knoop A., Kümler I. (2024). Real-world effectiveness of CDK 4/6 inhibitors in estrogen-positive metastatic breast cancer. BJC Rep..

[4] Arvold N.D., Taghian A.G., Niemierko A., Abi Raad R.F., Sreedhara M., Nguyen P.L., Bellon J.R., Wong J.S., Smith B.L., Harris J.R. (2011). Age, breast cancer subtype approximation, and local recurrence after breast-conserving therapy. J. Clin. Oncol..

[5] Huang J., Zheng L., Sun Z., Li J. (2022). CDK4/6 inhibitor resistance mechanisms and treatment strategies (Review). Int. J. Mol. Med..

[6] Pu D., Xu D., Wu Y., Chen H., Shi G., Feng D., Zhang M., Liu Z., Li J. (2024). Efficacy of CDK4/6 inhibitors combined with endocrine therapy in HR+/HER2-breast cancer: An umbrella review. J. Cancer Res. Clin. Oncol..

[7] Kappel C., Elliott M.J., Kumar V., Nadler M.B., Desnoyers A., Amir E. (2024). Comparative overall survival of CDK4/6 inhibitors in combination with endocrine therapy in advanced breast cancer. Sci. Rep..

[8] Thill M., Schmidt M. (2018). Management of adverse events during cyclin-dependent kinase 4/6 (CDK4/6) inhibitor-based treatment in breast cancer. Ther. Adv. Med. Oncol..

[9] Finn R.S., Crown J.P., Lang I., Boer K., Bondarenko I.M., Kulyk S.O., Ettl J., Patel R., Pinter T., Schmidt M. (2015). The cyclin-dependent kinase 4/6 inhibitor palbociclib in combination with letrozole versus letrozole alone as first-line treatment of oestrogen receptor-positive, HER2-negative, advanced breast cancer (PALOMA-1/TRIO-18): A randomised phase 2 study. Lancet Oncol..

[10] Finn R.S., Martin M., Rugo H.S., Jones S., Im S.A., Gelmon K., Harbeck N., Lipatov O.N., Walshe J.M., Moulder S. (2016). Palbociclib and Letrozole in Advanced Breast Cancer. N. Engl. J. Med..

[11] Turner N.C., Ro J., André F., Loi S., Verma S., Iwata H., Harbeck N., Loibl S., Huang Bartlett C., Zhang K. (2015). PALOMA3 Study Group. Palbociclib in Hormone-Receptor-Positive Advanced Breast Cancer. N. Engl. J. Med..

[12] Hortobagyi G.N., Stemmer S.M., Burris H.A., Yap Y.S., Sonke G.S., Paluch-Shimon S., Campone M., Blackwell K.L., André F., Winer E.P. (2016). Ribociclib as first-line therapy for HR-positive, advanced breast cancer. N. Engl. J. Med..

[13] Slamon D.J., Neven P., Chia S., Fasching P.A., De Laurentiis M., Im S.A., Petrakova K., Bianchi G.V., Esteva F.J., Martín M. (2018). Phase III randomized study of ribociclib and fulvestrant in hormone receptor-positive, human epidermal growth factor receptor 2-negative advanced breast cancer: MONALEESA-3. J. Clin. Oncol..

[14] Lu Y.S., Im S.A., Colleoni M., Franke F., Bardia A., Cardoso F., Harbeck N., Hurvitz S., Chow L., Sohn J. (2022). Updated overall survival of ribociclib plus endocrine therapy versus endocrine therapy alone in pre- and perimenopausal patients with HR+/HER2- advanced breast cancer in MONALEESA-7: A Phase III randomized clinical trial. Clin. Cancer Res..

[15] Sledge G.W., Toi M., Neven P., Sohn J., Inoue K., Pivot X., Burdaeva O., Okera M., Masuda N., Kaufman P.A. (2017). MONARCH 2: Abemaciclib in combination with fulvestrant in women with HR+/HER2- advanced breast cancer who had progressed while receiving endocrine therapy. J. Clin. Oncol..

[16] Goetz M.P., Toi M., Campone M., Sohn J., Paluch-Shimon S., Huober J., Park I.H., Trédan O., Chen S.C., Manso L. (2017). MONARCH 3: Abemaciclib as initial therapy for advanced breast cancer. J. Clin. Oncol..

[17] Ge I., Berner K., Mathis M., Hensgen C., Mayer S., Erbes T., Juhasz-Böss I., Asberger J. (2024). Real-world data analysis of cdk4/6 inhibitor therapy-a patient-centric single center study. Cancers.

[18] Low J.L., Lim E., Bharwani L., Wong A., Wong K., Ow S., Lim S.E., Lee M., Choo J., Lim J. (2022). Real-world outcomes from use of CDK4/6 inhibitors in the management of advanced/metastatic breast cancer in Asia. Ther. Adv. Med. Oncol..

[19] Miron A.I., Anghel A.V., Barnonschi A.A., Mitre R., Liscu H.D., Găinariu E., Pătru R., Coniac S. (2023). Real-World Outcomes of CDK4/6 Inhibitors Treatment in Metastatic Breast Cancer in Romania. Diagnostics.

[20] Harbeck N., Bartlett M., Spurden D., Hooper B., Zhan L., Rosta E., Cameron C., Mitra D., Zhou A. (2021). CDK4/6 inhibitors in HR+/HER2- advanced/metastatic breast cancer: A systematic literature review of real-world evidence studies. Future Oncol..

[21] Schneeweiss A., Ettl J., Lüftner D., Beckmann M.W., Belleville E., Fasching P.A., Fehm T.N., Geberth M., Häberle L., Hadji P. (2020). Initial experience with CDK4/6 inhibitor-based therapies compared to antihormone monotherapies in routine clinical use in patients with hormone receptor positive, HER2 negative breast cancer—Data from the PRAEGNANT research network for the first 2 years of drug availability in Germany. Breast.

[22] Piezzo M., Chiodini P., Riemma M., Cocco S., Caputo R., Cianniello D., Di Gioia G., Di Lauro V., Rella F.D., Fusco G. (2020). Progression-free survival and overall survival of CDK 4/6 inhibitors plus endocrine therapy in metastatic breast cancer: A systematic review and meta-analysis. Int. J. Mol. Sci..

[23] Das Majumdar S.K., Barik S.K., Pattanaik A., Das D.K., Parida D.K. (2024). Role of cyclin-dependent kinase 4/6 in metastatic breast cancer: Real-world data from a tertiary care institute in Eastern India. Cureus.

[24] Syed Y.Y. (2017). Ribociclib: First Global Approval. Drugs.

[25] Im S.A., Lu Y.S., Bardia A., Harbeck N., Colleoni M., Franke F., Chow L., Sohn J., Lee K.S., Campos-Gomez S. (2019). Overall Survival with Ribociclib plus Endocrine Therapy in Breast Cancer. N. Engl. J. Med..

[26] Finn R.S., Boer K., Bondarenko I., Patel R., Pinter T., Schmidt M., Shparyk Y.V., Thummala A., Voitko N., Bananis E. (2020). Overall survival results from the randomized phase 2 study of palbociclib in combination with letrozole versus letrozole alone for first-line treatment of ER+/HER2- advanced breast cancer (PALOMA-1, TRIO-18). Breast Cancer Res. Treat..

[27] Witkiewicz A.K., Schultz E., Wang J., Hamilton D., Levine E., O’Connor T., Knudsen E.S. (2023). Determinants of response to CDK4/6 inhibitors in the real-world setting. NPJ Precis. Oncol..

